# Development and psychometric evaluation of the High-Risk Pregnancy Well-Being Index in Mashhad: a methodological study

**DOI:** 10.1186/s12978-022-01529-0

**Published:** 2022-12-01

**Authors:** Kobra Mirzakhani, Talat Khadivzadeh, Farhad Faridhosseini, Abbas Ebadi

**Affiliations:** 1 Nursing and Midwifery Care Research Center, Mashhad University of Medical Sciences, Mashhad, Iran; 2 Department of Midwifery, School of Nursing and Midwifery, Mashhad University of Medical Sciences, Mashhad, Iran; 3 Psychiatry and Behavioral Sciences Research Center, Mashhad University of Medical Sciences, Mashhad, Iran; 4 Behavioral Sciences Research Center, Life Style Institute, Baqiyatallah University of Medical Sciences, Tehran, Iran; 5 Research Center for Life & Health Sciences & Biotechnology of the Police, Direction oh Health, Rescue & Treatment, Police Headquarter, Tehran, Iran

**Keywords:** Well-being, High-risk pregnancy, Instrument, Psychometric, Evaluation, Well-Being Index

## Abstract

**Background:**

Assessment of well-being in high-risk pregnancy (HRP) is the key to achieve positive maternal and fetal outcomes. Although there are a wide range of instruments for well-being assessment, none of them is comprehensive for well-being assessment in HRP. The present study aimed at the development and psychometric evaluation of the High-Risk Pregnancy Well-Being Index (HRPWBI).

**Methods:**

This methodological study was conducted using the Waltz’s four-step method. The dimensions of well-being in HRP were determined based on a conceptual model and the blueprint and the item pool of HRPWBI were developed. Then, the face and the content validity were assessed and item analysis was performed. Construct validity was also assessed through exploratory factor analysis with principal component analysis on the data obtained from 376 women with HRP in Mashhad, Iran. Finally, internal consistency, test–retest stability, sensitivity, and interpretability of HRPWBI were assessed.

**Results:**

The scale- content validity index (SCVI) of HRPWBI was 0.91. In factor analysis, 33 items were loaded on seven factors which explained 53.77% of the total variance. Internal consistency, relative stability, absolute stability, sensitivity, and interpretability of HRPWBI were confirmed with a Cronbach’s alpha of 0.84, a test–retest intraclass correlation coefficient of 0.97, a standard error of measurement of 0.92, a minimal detectable change of 8.09, and a minimal important change of 2.92, respectively.

**Conclusion:**

HRPWBI is a valid and reliable instrument for well-being assessment among women with HRP. It can be used to assess well-being and the effects of well-being improvement interventions on well-being among women with HRP.

## Plain Language Summary

High-risk pregnancy (HRP) is one of the world’s most serious reproductive health issues. Assessing well-being in high-risk pregnancies is the key to achieving positive maternal and fetal outcomes. This study aims to develop, psychometrically test and validate the High-Risk Pregnancy Well-Being Index (HRPWBI). This study demonstrated that the (HRPWBI) is a valid and reliable tool for assessing the well-being of HRP women. It can evaluate the state of well-being in women with high-risk pregnancies, investigate the factors influencing the well-being of mothers with high-risk pregnancies, and examine the effects of interventions on improving well-being in women with HRP. To ensure that Policy maker, researchers and Clinicians in management of women with High-Risk Pregnancy incorporates measures to improve the well-being of this vulnerable group of women with high-risk pregnancies into its planning and policies.

## Background

High-risk pregnancy (HRP) is one of the main reproductive health challenges throughout the world [1]. By definition, HRP is a pregnancy in which the pregnant woman or her fetus is at risk for injury [2, 3]. The leading causes of HRP are previous physical or mental problems or new problems developed during pregnancy [4]. The prevalence of HRP in different countries varies from 6 to 40.5% [1, 5] and is 39.8–75.6% in different areas of Iran [6, 7]. Each day, HRP leads to the deaths of 800 pregnant women in the world [8].

HRP is associated with many different consequences. Physical changes in HRP can lead to mood and social changes in pregnant women [9, 10]. Compared with women with low-risk pregnancy, woman with HRP more frequently experience restlessness, fear, loss of control, disability, anger, anxiety, despair, and feeling of guilt [11] and less frequently experience positive feelings such as self-confidence, despair, eagerness, happiness, and pleasure [12]. Therefore, HRP is associated with high levels of uncertainty, mental strains, and ailment [13, 14]. A study reported that the prevalence of mental disorders in HRP was 22.7–36.6% for depression, 17.3–27.3% for anxiety, and 19.8–31.7% for stress [15]. Negative feelings in pregnancy may lead to inappropriate or risky decisions. For example, a meta-analysis reported greater risk of suicide among women with HRP [16]. HRP and its associated physical, mental, and social consequences can negatively affect afflicted women’s well-being [1].

Well-being and well-being in HRP are broadly defined as having senses of pleasure, happiness, and satisfaction and consists of physical, functional, emotional, intellectual, psychological, familial, and social aspects [11]. Well-being improvement and distress reduction during HRP are the keys to achieve positive maternal–fetal outcomes [17–19]. Therefore, besides physical problems, healthcare providers need to pay close attention to the feelings, satisfaction, and well-being of women during HRP and need to employ appropriate interventions to reduce their problems and improve their well-being [20, 21].

One of the most essential prerequisites to well-being improvement in HRP is its careful assessment using valid and reliable instruments. There are many different instruments for the assessment of the different aspects of well-being. However, these instruments were developed based on various approaches to well-being and have various items on the different aspects of well-being [22, 23]. and hence, there is no comprehensive instrument or approach for the assessment of all aspects of well-being [24]. Some previous studies considered lack of depression, anxiety, and psychological disorders as well-being and used depression- and anxiety-related instruments for well-being assessment [23, 25–28]. Some studies also equated well-being with acceptable physical health and hence, used physical health assessment instruments for well-being assessment [29, 30]. Some other studies also used instruments on general psychological well-being for well-being assessment among women with HRP [31, 32]. Such instruments are useful for well-being assessment among the general public but not useful for women with HRP. For example, The Well-Being 5 instrument was developed for the assessment of well-being in communities, has no items on the cognitive and the psychological aspects of well-being [33] and hence, is not valid for well-being assessment among women with HRP. The Ryff Scale of Psychological Well-Being and the Subjective Well-being Scale also assess psychological and subjective well-being and have items which address general well-being [34, 35]. Since well-being is affected by the immediate physical and mental conditions [36], instrument for general well-being assessment may not be valid for well-being assessment among patients or women with HRP. Therefore, comprehensive instruments for the comprehensive assessment of well-being in HRP are needed. The present study aimed at the development and psychometric evaluation of the High-Risk Pregnancy Well-Being Index (HRPWBI).

## Methods

This methodological study was conducted by using the Waltz’s method. The four steps of this method are selection of a conceptual model to delineate the nursing or healthcare aspects of the measurement process, determination of the objectives of the measurement, development of a blueprint, and development of the instrument [37].

1. Selection of a conceptual model: The conceptual model of the present study was developed based on an integrative literature review into well-being in HRP [11] and a qualitative study into the experiences of well-being in HRP [38].
2. Determination of the objectives of the measurement: The objectives of the measurement in the present study were the attributes or the dimensions of the concept of well-being in HRP determined in our concept analysis studies [11, 38]. The method of measurement was determined to be self-report and the level of measurement was determined to be a Likert scale with the five points of “Always”, “Often”, “Sometimes”, “Rarely”, and “Never”.
3. Development of a blueprint: In this step, the appropriate number of the items for each objective of the measurement was determined.
4. Development of the instrument: A large pool of 219 items was generated based on the codes generated in our concept analysis studies [11, 38]. Then, overlapping items were combined and repetitive items were excluded and the number of items reached 138.

### Psychometric evaluation

The psychometric properties of HRPWBI, namely face validity, content validity, construct validity, and reliability, were assessed. Study setting for psychometric evaluation consisted of HRP care wards and clinics of Qaem, Imam Reza, and Ommolbanin public hospitals and Mehr and Pasteur private hospitals as well as the Comprehensive Healthcare Center number 3 in Mashhad, Iran.

### Face validity evaluation

Face validity was evaluated using qualitative and quantitative methods. In qualitative evaluation of face validity, face-to-face interviews were held with ten women with HRP to ask them to comment on the difficulty, appropriateness, and ambiguity of each HRPWBI item. In quantitative face validity evaluation, impact score was calculated for each item. Accordingly, the same ten women were asked to rate the importance of each item on a five-point scale as follows: 5: “Completely important”; 4: “Important”; 3: “Relatively important”; 2: “Somewhat important”; and 1: “Not important”. Then, item impact score was calculated through multiplying frequency (%) by importance. Items with item impact scores more than 1.5 were considered acceptable [39].

### Content validity evaluation

Content validity was evaluated using qualitative and quantitative methods. In qualitative evaluation of content validity, fifteen experts in psychology (n = 2), psychiatry (n = 1), gynecology (n = 2), reproductive health and midwifery (n = 5), instrument development (n = 3), and concept analysis (n = 2) assessed the grammar, wording, allocation, and scaling of the items. In quantitative content validity evaluation, content validity ratio (CVR) and content validity index (CVI) were calculated. Accordingly, the same experts were asked to determine item essentiality on a three-point scale as “Essential”, “Useful but not essential”, and “Not essential”. Then, CVR was calculated using the (*n*_*e*_*–N*/2)/(*N*/2) formula, where *n*_*e*_ was the number of experts rated the item essential and *N* was the total number of experts. Based on the Lawshe’s critical value for CVR, the minimum acceptable CVR for fifteen experts is 0.49 [40]. Moreover, seventeen experts in psychology (n = 2), psychiatry (n = 1), gynecology (n = 2), reproductive health and midwifery (n = 7), instrument development (n = 3), and concept analysis (n = 2) rated item relevance as follows: 1: “Not relevant”; 2: “Somewhat relevant”; 3: “Relevant but needs revision”; and 4: “Relevant”. Their rating scores were used to calculate item CVI (I-CVI) through dividing the number of experts who had rated the item 3 or 4 by the total number of the experts. Items with CVI values more than 0.78 were considered appropriate, items with CVI values 0.7–0.78 were revised, and items with CVI values less than 0.7 were considered unacceptable and were excluded [41]. Scale CVI (S-CVI) was also calculated for the whole HRPWBI.

To reduce the risk of chance agreement, Cohen’s Kappa coefficient was calculated through the following formula: (*I-CVI* *−* *Pc*)/(1 *−* *Pc*). *Pc* was calculated through the following formula: (*N*/*A* × (*N* *−* *A*)) × 0.5^*N*^, where *A* was the number of experts agreed on item relevance and *N* was the total number of experts. Kappa values more than 0.74 were considered acceptable [42].

### Item analysis

Item analysis was performed in a pilot study on 44 women with HRP diagnosed according the NICE guideline [4]. Participants completed HRPWBI and their data were used for item analysis through the Loop method. Cronbach’s alpha values more than 0.70, inter-item correlation coefficients between 0.3 and 0.7, and item-scale correlation coefficients more than 0.2 were considered acceptable [43].

### Construct validity evaluation

Construct validity was evaluated using exploratory factor analysis. Accordingly, 376 women with HRP diagnosed according the NICE guideline [4] were selected, provided with information about the study aim, ensured that their data would remain confidential, and asked to sign the informed consent form of the study and complete HRPWBI. Then, the SPSS software (v. 25.0) was used to perform exploratory factor analysis. Data normality was tested through skewness (± 3) and kurtosis (± 7) measures and factorability was tested using the Keyser–Meyer–Olkin statistic and the Bartlett’s test. Missing values were replaced with the mean score, outliers were corrected, and the linearity of the relationship among the variables was tested through testing whether the items had communalities more than 0.4 [44]. The principal component analysis was used in exploratory factor analysis. The three main steps of this analysis are calculation of the correlation matrix of all variables (acceptable coefficients are in the range of 0.3–0.7), extracting the primary factors, and rotating the extracted factors. The number of extractable factors was determined using eigenvalue, scree plot, and the theoretical knowledge obtained from our concept analysis studies [42, 44]. The rotation of the extracted factors was performed to determine the best factor loading and create interpretable factors. Varimax rotation provided the best factor loading with the lowest cross-loading and the best interpretability [45].

### Reliability evaluation

In reliability evaluation, 53 women with HRP twice completed HRPWBI with a 10-day interval [45]. Ten participants were excluded due to significant changes in their pregnancy (n = 7) or reluctance to re-complete HRPWBI at retest. Internal consistency was assessed by calculating Cronbach’s alpha and theta and values more than 0.7 were interpreted as acceptable internal consistency. Test–retest data were used to determine relative and absolute stability. Relative stability was assessed by calculating intraclass correlation coefficient (ICC) [42] and absolute stability was assessed by calculating standard error of measurement (SEM) through the ${SEM}_{agreement}=SD \times \sqrt{\left(1-{ICC}_{agreement}\right)}$ formula. *SD* in this formula was the standard deviation of the test–retest scores.

Responsiveness and sensitivity of HRPWBI were evaluated through the Consensus-based Standards for the Selection of health Measurement Instrument (COSMIN). Accordingly, the smallest detectable change (SDC) or minimal detectable change (MDC) was calculated through the $MDC=1.96 \times \sqrt {2\times SEM}$ formula [45]. MDC% was also calculated through the $MDC\%=\left(MDC-mean\right)\times 100$ formula. In this formula, the pretest and the posttest mean scores of HRPWBI and its subscales were used as mean and thereby, MDC% of HRPWBI and all it subscales were calculated. MDC% values less than 30 were considered acceptable and MDC% values less than 10 were considered excellent [45, 46].

The interpretability of HRPWBI was determined through calculating the minimal important change (MIC), percentage of missing values, and floor and ceiling effects. MIC was calculated through the $MIC=0.5\times SD\;of\;\Delta score$ formula.

### Scoring

Items were scored on a five-point scale as follows: 5: “Always”; 4: “Often”; 3: “Sometimes”; 2: “Rarely”; and 1: “Never”. Items with negative wording were reversely scored. The raw scores of HRPWBI and its subscales were transformed to the 0–100 scale through linear transformation using the following formula [47],

$$Score=\frac{Raw\;score-Lowest\;raw\;score}{Highest\;raw\;score-Lowest\;raw\;score}\times 100$$

### Ethical considerations

The Ethics Committee of Mashhad University of Medical Sciences, Mashhad, Iran, approved this study (code: IR.MUMS.NURSE.REC.1397.039). Study aims were explained for participants and their written informed consent was obtained.

## Results

The primary HRPWBI was developed with 138 items and then, its psychometric properties were evaluated.

### Psychometric evaluation

#### Face and content validity evaluation

In face validity evaluation, no item was omitted and six items were revised. In content validity evaluation, 44 items were omitted because their CVR values were less than 0.49 and two items were omitted because their I-CVI values were less than 0.70. Experts also noted that fourteen items were overlapping and hence, they were either omitted or combined. Accordingly, the number of items reached 78 and the S-CVI of the 78-item HRPWBI was 0.91. The Cohen’s kappa coefficients of the items were 0.76–1, denoting acceptable content validity [41].

#### Item analysis

In the item analysis of the 78-item HRPWBI, six items had inter-item correlation coefficients more than 0.7, denoting their similarity or conceptual overlap. These six items were either omitted or combined. Moreover, the item-scale correlation coefficients of fourteen items were less than 0.2. Finally, seventeen items were omitted in this step and the 61-item HRPWBI was evaluated for its construct validity.

#### Construct validity evaluation

*Participants’ characteristics:* Participants were 376 women with HRP with an age mean of 30.48 ± 6.7 years in the range of 16–47. Thirty participants (8.1%) were in their first trimester, 109 participants (29.4%) were in their second trimester, and 237 participants (62.5%) were in their third trimester. Moreover, 103 participants (27.4%) were in their first pregnancy, 171 participants (45.5%) were in their second or third pregnancy, and 102 participants (27.1%) were in their fourth pregnancy or more. The most common causes of HRP were diabetes mellitus (n = 71; 18.88%), cardiovascular disease (n = 50; 13.3%), and hypertension (n = 48; 12.9%) (Table 1).

**Table 1:** The sociodemographic and clinical characteristics of the participants

Variable	Number%	Mean (SD)
All participants	n = 376	
Age	n = 376	30.48 ± 6.7
Educational level	Primary school: 89 (23.7)	
	Secondary school: 210 (55.8%)	
	University: 77 (20.5%)	
Occupation	Housewife: 327 (87%)	
	Student: 15 (4%)	
	Employed: 34 (9%)	
Gestational age	First trimester: 30(8.1%)	29.02 ± 9.07
	Second trimester: 109 (29.4%)	
	Third trimester: 237 (62.5%)	
Number of pregnancies	1: 103 (27.4%)	
	2: 87 (23.1%)	
	3: 84 (22.3)	
	4: 58 (15.4%)	
	5: 27 (7.2%)	
	6: ≥ 17 (4.6%)	
Causes of HRP^a^	Diabetes mellitus: 71 (18.88%), cardiovascular disease: 50 (13.3%)	
	Hypertension: 48 (12.9%)	
	Placenta Previa: 20 (5.3%)	
	PROM: 19 (5.1%)	
	Placenta Acreta: 18 (4.8%)	
	Thrombocytopenia: 12 (13.5%)	
	Kidney or liver or brain-nerve or skin or respiratory disorders: 20 (5.3%)	
	Other: 118 (21%)	

^a^High-risk pregnancy

*Normality testing* The distribution of the scores of 57 items (93.44%) was normal, while the mean scores of items 14, 44, and 59 were not normal based on their skewness and the mean scores of items 14, 44, 58, and 59 were not normal based on their kurtosis. The non-normal distribution of these items was controlled through assessing floor and ceiling effects [42].

*Missing values* Missing values in each item were less than 1% and were replaced with the mean score of the item.

*Correlation* Items 1, 3, 4, and 15 had no correlation with any other item at a coefficient of more than 0.3 and hence, were omitted [44]. None of the items had a strong correlation with other items at a coefficient of more than 0.7.

*Factorability* The Keyser–Meyer–Olkin statistic was 0.806 and the Bartlett’s test was statistically significant, confirming sampling adequacy and appropriate factor analysis model.

*Communalities of items* The table of communalities was assessed after performing principal component analysis and loading the items on their relevant factors. Items with the lowest communalities (i.e., outlier variables) were omitted. After omitting each item with the lowest communality, factor analysis was re-performed and finally, six items (i.e., items 7, 16, 17, 26, 27, and 35) were omitted and items with communalities more than 0.4 were accepted and kept [44].

*Primary factor extraction* Nine items had eigenvalues equal to 1 or more. Scree plot also revealed seven factors for HRPWBI. Each of these seven factors explained more than 5% of the variance and the cumulative variance was 53.78 (Table 2).

**Table 2:** Factors, items, and item factor loading values in HRPWBI

Factors	No.	No.	Item	Factors						
				1	2	3	4	5	6	7
Subjective well-being	1	Q8	I have fear over adverse events for my child constantly think about it	0.653						
	2	Q10	I fear more when I see critically-ill women	0.650						
	3	Q9	I have fear over unpredicted events during labor	0.635						
	4	Q11	I have developed stress due to pregnancy-related problems	0.597						
	5	Q12	I have stress over losing my fetus	0.579						
	6	Q13	I feel sadness and grief	0.576						
	7	Q5	I feel worried	0.570						
	8	Q20	I feel guilty if any distress or complication happens for my fetus	0.549						
	9	Q21	I blame myself for the problems in the pregnancy	0.536						
	10	Q19	I feel lonely	0.458						
Marital well-being	11	Q52	My husband understands me in pregnancy		0.822					
	12	Q55	My husband’s sense of responsibility calms me		0.764					
	13	Q53	I have trust in the durability of my marital relationships		0.745					
	14	Q54	My husband helps me in household activities		0.698					
	15	Q51	I’m satisfied with my marital relationships in pregnancy		0.622					
Self-efficacy and independence	16	Q37	I can independently go to the toilet			0.813				
	17	Q38	I can perform my daily activities			0.768				
	18	Q40	I can independently perform my social tasks			0.741				
	19	Q36	I can stand on my feet and walk			0.717				
	20	Q42	I can appropriately care for myself			0.631				
Social well-being	21	Q49	Others’ curiosity about my pregnancy annoys me				0.722			
	22	Q48	I hide my pregnancy from others as much as possible				0.673			
	23	Q31	I’m worried about others’ awareness of my pregnancy-related problems				0.596			
	24	Q50	Others think that I am an ill and disabled woman				0.588			
Perceived well-being about healthcare services	25	Q47	Healthcare providers empathize with me					0.747		
	26	Q46	Healthcare providers provide me with adequate information					0.745		
	27	Q34	I trust the established diagnosis and healthcare services					0.628		
Health anxiety	28	Q23	I am worried about my own health						0.809	
	29	Q24	I am worried about the long-term complications of high-risk pregnancy						0.658	
	30	Q30	I am worried about timely access to physician or hospital						0.467	
Spiritual well-being	31	Q58	The ability to perform religious practices gives me the good feeling of health							0.799
	32	Q59	I am sure that God protects the health of me and my child							0.775
	33	Q57	I feel worthy in pregnancy							0.491
			Variance	11.37%	10.06%	8.89%	6.99%	5.55%	5.50%	5.38%
			Total variance	53.77%						

High-Risk Pregnancy Well-Being Index

*Factor extraction method* Factors were extracted through the principal component analysis. Items with high correlation with each other were grouped into a factor and the minimum acceptable factor loading was considered to be 0.4. Items which were not loaded on any factor were omitted and principal component analysis was re-performed. Accordingly, seventeen items (i.e., items 2, 6, 14, 22, 28, 29, 32, 33, 39, 41, 43, 44, 45, 56, 60, and 61) were omitted because they were not loaded on any factor.

*Rotation of the extracted factors* Varimax rotation provided the best factor loading with the lowest cross-loading and the best interpretation. Item 25 (“I am concerned with the long-term effects of HRP on the child”) was loaded on both the first and the fifth factors with factor loading values more than 0.4 and factor loading difference of less than 0.2, denoting cross-loading. Therefore, this item was omitted. Finally, the 33 remaining items were loaded on seven factors. Each factor explained more than 5% of the variance and all factors explained 53.77% of the total variance. Extracted factors had acceptable comprehensibility and interpretability and adequate number of items (three or more) and their factor loading values were high (more than 0.4) (Table 2).

*Labeling of the factors* Factors were labeled based on their items, particularly items with the highest factor loading values (Table 2). The seven extracted factors of HRPWBI were subjective well-being, marital well-being, self-efficacy and independence, social well-being, perceived well-being about healthcare services, health anxiety, and spiritual well-being.

#### Reliability evaluation

The Cronbach’s alpha of HRPWBI was 0.84 and the theta coefficients of HRPWBI and all its factors were more than 0.7 (Table 3), confirming acceptable internal consistency. The ICC and SEM of HRPWBI were 0.97 and 2.92 (Table 3), which confirm the acceptable relative and absolute stability of the index, respectively.

**Table 3:** Internal consistency and relative and absolute stability of HRPWBI

No.	Factor	Alpha	Theta	ICC	95% CI for ICC	SEM	P value
1	Subjective well-being	0.866	0.814	0.965	0.935–0.981	1.773	< 0.001
2	Marital well-being	0.817	0.875	0.95	0.907–0.973	0.776	< 0.001
3	Self-efficacy and independence	0.865	0.825	0.971	0.946–0.984	0.878	< 0.001
4	Social well-being	0.702	0.758	0.919	0.85–0.956	1.19	< 0.001
5	Perceived well-being about healthcare services	0.644	0.788	0.909	0.833–0.951	0.8	< 0.001
6	Health anxiety	0.675	0.799	0.948	0.903–0.972	0.77	< 0.001
7	Spiritual well-being	0.873	0.799	0.974	0.952–0.986	0.42	< 0.001
8	Total	0.837		0.974	0.951–0.986	2.92	< 0.001

High-Risk Pregnancy Well-Being Index

#### Responsiveness and interpretability

The MDC, MDC%, and SEM of HRPWBI were respectively 8.09, 6.63, and 2.92 and MIC was less than MDC (Table 4), confirming the acceptable responsiveness of the index.

**Table 4:** SEM, MDC, MDC%, MIC, and Kappa values to assess the test–retest absolute stability and agreement of HRPWBI

No.	Factors	Score range	Mean ± SD	SEM	MDC	MIC	Kappa	MDC%	Result
1	Subjective well-being	10–50	31.44 ± 9.85	1.773	4.9	1.82	+	15.58	Acceptable
2	Marital well-being	5–25	22.18 ± 4.54	0.776	2.14	0.77	+	10.36	Acceptable
3	Self-efficacy and independence	5–25	22.75 ± 4.36	0.878	2.43	0.86	+	10.68	Acceptable
4	Social well-being	4–20	15.58 ± 4.22	1.19	3.29	1.14	+	21.11	Acceptable
5	Perceived well-being about healthcare services	3–15	12.38 ± 2.54	0.8	2.21	1.14	+	17.85	Acceptable
6	Health anxiety	3–15	7.63 ± 3.53	0.77	2.13	0.79	+	27.91	Acceptable
7	Spiritual well-being	3–15	13.8 ± 1.45	0.42	1.21	0.41	+	8.76	Excellent
8	Total	33–165	121.85 ± 16.62	2.92	8.09	2.92	+	6.63	Excellent

High-Risk Pregnancy Well-Being Index

#### Roof and ceiling effects

None of the participants obtained the lowest possible score of HRP (i.e., 33) and only one participant (0.3%) obtained its highest possible score (i.e., 165). Therefore, the roof effect was zero and the ceiling effect was 0.3%. Floor and ceiling effects less than 15% are acceptable.

#### Easy applicability

The easy applicability of HRPWBI was assessed through measuring the amount of time needed for its answering which was 6–18 min with a mean of 11.1 ± 1.96. Moreover, missing values of each item were less than 1%. Therefore, the easy applicability of HRPWBI is confirmed.

#### Scoring

Items are scored 1–5 and hence, the possible total score of the 33-item HRPWBI is 33–165.

## Discussion

Study findings showed that the final HRPWBI is a valid and reliable instrument with 33 items in seven dimensions. All items had acceptable factor loading, indicating their significant effects on the concept of HRP. Moreover, all items had acceptable correlation with their corresponding factors. Relative and absolute stability of the instrument were also confirmed and the instrument had low SEM, acceptable responsiveness, acceptable interpretability, and easy applicability. Construct validity evaluation showed that HRPWBI had seven factors which were labeled subjective well-being, marital well-being, self-efficacy and independence, social well-being, perceived well-being about healthcare services, health anxiety, and spiritual well-being.

### Subjective well-being

The subjective well-being factor had ten items and its explained variance was more than other factors. The items of this factor are on mood-related, mental, and emotional aspects of HRP such as feelings of fear, stress, anxiety, grief, guilt, self-blame, and loneliness. The origin of these feelings is usually the conditions of fetus and pregnancy [11, 48]. The questionnaire developed by Rasmussen et al. for the measurement of pregnancy and postnatal well-being among women with type 1 diabetes mellitus has fifteen items in its psychological well-being dimension which are similar to the items in the subjective well-being dimension of HRPWBI [49]. However, Rasmussen et al. did not assess construct validity of their questionnaire through factor analysis and hence, the psychological well-being dimension of their questionnaire had items on self-efficacy, self-control, and self-confidence for diabetes management in pregnancy, while the subjective well-being dimension of HRPWBI is clearly distinguishable from the self-efficacy and independence dimension.

### Marital well-being

The marital well-being dimension of HRPWBI had the second rank respecting the amount of the explained variance. It has five items on husband’s understanding of HRP, trust in husband, help by husband, and satisfaction with marital relationship. This is in line with the findings of our previous qualitative study into the factors affecting marital well-being in HRP [48]. To the best of our knowledge, none of the existing well-being assessment instruments include a marital well-being dimension [22]. Most of these instruments such as the Ryff Scale of Psychological Well-Being, have an interpersonal relationships dimension. However, our findings revealed that compared with other types of interpersonal relationships, marital relationships had greater effects on well-being in HRP and hence, were identified in factor analysis as a main dimension of HRPWBI. The questionnaire of Rasmussen et al. also has items on marital relationships. However, those items are mostly on husband’s support in the postpartum period and for child care [50] and therefore, are not valid for well-being assessment in HRP. Moreover, the construct validity of that instrument was not assessed to determine whether it has an independent marital well-being dimension.

### Self-efficacy and independence

The third dimension of HRPWBI was self-efficacy and independence which refers to the feeling of self-efficacy and ability to do physical, social, and self-care activities. Ryff Scale of Psychological Well-Being also has items on independence and autonomy [35] but its target population is the general public and hence, is not appropriate for women with HRP. It is noteworthy that the most usual activities of daily living, such as going to the toilet, may be difficult or risky for women with HRP [48]. The environmental mastery dimension of the Ryff Scale of Psychological Well-Being questionnaire may also refer to self-efficacy and independence [35]. However, its items are not relevant to well-being in HRP and hence, are not valid for well-being assessment among women with HRP.

### Social well-being

Social well-being was the fourth dimension of HRPWBI and had the fourth rank respecting the amount of the explained variance. The four items of this dimension are on feelings of objective relationships with the society. Well-being is a personal experience which deals mostly with personal satisfaction and positive emotions. Nonetheless, humans are social beings and encounter different social challenges and hence, their feelings and emotions are affected by social life [51]. Accordingly, Keyes introduced the concept of social well-being and noted that social well-being refers to the personal report of the quality of relationships with the others and the surrounding environment [52]. Keyes’ Subjective Well-being Scale has items on individuals’ social responsibilities and their interactions with society. Nonetheless, that scale was developed for well-being assessment among the general public [34] and its items are not valid for well-being assessment among women with HRP [11, 48]. The Quality of Well-Being Self-Administered Scale also has a social activity and self-care dimension with items on independence and self-care [53]. However, the social well-being dimension of HRPWBI refers to interpersonal relationships and interactions. The items of the positive relations with others dimension of the Ryff Scale of Psychological Well-Being [35] are also not essential for well-being assessment among women with HRP [11, 48].

### Perceived well-being about healthcare services

The fifth dimension of HRPWBI was perceived well-being about healthcare services, which refers to women’s feeling of well-being with respect to healthcare providers’ empathetic relationships and their quality information for women to gain their trust in diagnosis and healthcare services. Although most instruments on well-being measurement, such as the Ryff Scale of Psychological Well-Being, have dimensions on interpersonal relationships [35], their items do not address trust in diagnosis and healthcare services for women with HRP and hence, do not provide reliable information about women’s trust in healthcare services.

### Health anxiety

Health anxiety was the sixth dimension of HRPWBI. This dimension refers to the concerns and worries of women with HRP concerning HRP and access to healthcare services. The questionnaire of Rasmussen et al. also includes a dimension about women’s concerns over their physical well-being and their fetus’ which is somewhat similar to the health anxiety dimension of HRPWBI. However, that dimension has no item on access to healthcare services [50]. The Well-Being Index also has items on access to thirteen types of essential general services such as food, water, money, shelter, and safety. This index is appropriate for well-being assessment among the general public and is not specific to women with HRP [54].

### Spiritual well-being

Spiritual well-being, the seventh dimension of HRPWBI, refers to the fact that well-being in HRP depends on trust in God’ protection of health as well as having good feelings about the ability to perform religious practices. Studies show that religious beliefs have significant effects on well-being. For example, a study reported that 86% of Americans considered religious and spiritual beliefs important to their well-being [54]. The items of the Spiritual Well-Being questionnaire are also similar to the items of the spiritual well-being dimension of HRPWBI. However, that questionnaire is specific to spiritual well-being and cannot be used for comprehensive well-being assessment among women with HRP [55], while HRPWBI is a comprehensive instrument with items on different aspects of well-being in HRP.

Our integrative review [11] and qualitative study [48] had shown that well-being in HRP had a physical dimension. However, our construct validity evaluation in the present study showed no physical well-being dimension for HRPWBI. Some well-being assessment instruments such as the Personal Well-Being Index [56], the Quality of Well-Being Scale, and the Quality of Well-Being Self-Administered Scale actually assess health-related quality of life and have many items on the physical aspect of well-being or quality of life [53]. The Well-Being Index also focuses on the assessment of health and the physical aspect of well-being because its developers believed that well-being is in line with health [54]. The Well-Being 5 questionnaire also includes a physical dimension with items on health status, health-related behaviors, and drug abuse [33, 54]. On the other hand, most well-being assessment instruments such as the Warwick-Edinburgh Mental Well-Being Scale focus just on the psychological aspect of well-being [57]. The twelve-item Well-Being Questionnaire [58], the Subjective Well-being Scale [34], and the Ryff Scale of Psychological Well-Being [35] also assess psychological or subjective well-being. Some well-being theorists believe that well-being is a subjective concept associated with inner happiness and energy and has no physical dimension [59]. The inclusion of the physical dimension of well-being in our integrative review [11] and qualitative study [48] and its exclusion from HRPWBI may denote that despite the importance of physical health status in determining well-being among women with HRP, other dimensions of well-being in HRP are more important and share more contribution to its variance. Nonetheless, available clinical guidelines for care delivery to women with HRP mostly address physical health and management of physical problems and pay limited attention, if any, to other aspects of well-being. HRPWBI was developed based on the standard principles of instrument development and hence, can be used to measure the concept of well-being in HRP. A main limitation of the present study was the potential effects of participants’ conditions on their responses to HRPWBI. This index is only for women with high-risk pregnancies. A psychometric test is required for low-risk pregnancies. A further study is needed to analyze the confirmation factor in women with high-risk and low-risk pregnancies, as well as in other contexts. Women with high-risk pregnancies can be studied using this tool to describe their state of well-being and to identify factors influencing well-being. A descriptive study uses this tool to compare the well-being of women during high-risk and low-risk pregnancies. Clinical trials can also assess the effectiveness of interventions in improving well-being during high-risk pregnancies and suggest appropriate measures to policymakers and planners based on their findings.

## Conclusion

HRPWBI is a valid and reliable instrument for well-being assessment among women with HRP. Based on the data obtained by this instrument, interventions can be developed to improve well-being among women with HRP. Moreover, the instrument can be used to assess the effects of such interventions. Future studies can use HRPWBI for well-being assessment among women with HRP.

## Data Availability

The data collected and analyzed in the present study are available upon reasonable request from the corresponding author (Ebadi1347@yahoo.com) without jeopardizing participant confidentiality.

## Abbreviations

HRP: High-risk pregnancy
HRPWBI: High-Risk Pregnancy Well-Being Index
